# Commentary: A BK (Slo1) channel journey from molecule to physiology

**DOI:** 10.3389/fphar.2017.00188

**Published:** 2017-04-05

**Authors:** Domenico Tricarico, Antonietta Mele

**Affiliations:** Department of Pharmacy-Drug Science, University of BariBari, Italy

**Keywords:** calcium activated potassium channel, skeletal muscle, splicing isoforms, periodic paralysis, acetazolamide, dichlorphenamide

Prof. Ramon Latorre of Centro Interdisciplinario de Neurociencia de Valparaíso, Facultad de Ciencias, Universidad de Valparaíso, Chile, and co-authors in their review paper deal with the hallmarks of big Ca^2+^-activated K^+^ (BK) channel biophysics and its physiological impact on specific cells and tissues, highlighting its relationship with auxiliary subunit expression (Contreras et al., 2013). However, the molecular aspects and role of skeletal muscle BK channel subtypes were not extensively discussed. One of the scientific programs running in our laboratories is related to the role of BK channel in native skeletal muscle fibers using patch-clamp in excised patch mode and molecular biology techniques. Briefly, in skeletal muscle the opening of BK channel triggered by depolarization and Ca^2+^ ions increases the duration of the hyperpolarization phase between bursts of action potentials reducing the firing capability during discharge.

One important aspect concerns the BK channel diversity in the tissues. In fact, the functional diversity of BK channel is established by the association of the alpha subunit encoded by *KCNMA1* gene with auxiliary β1–β4 subunits encoded by *KCNMB1–4* genes with the contribution of novel γ subunits (Contreras et al., 2013; Toro et al., 2014; Torres et al., 2014). In skeletal muscle we established that the alternative splicing of the *KCNMA1/slo1* gene is the main mechanism regulating BK channel diversity in the muscle phenotypes (Shipston, 2001; Tricarico et al., 2005; Dinardo et al., 2012). Slow-twitch rat fibers show an elevated expression/activity of BK channel which is characterized by a low sensitivity to Ca^2+^ ions and absence of response to BK channel openers such as acetazolamide (Tricarico et al., 2004, 2005). In contrast, BK channel of fast-twitch rat fibers show a low expression/activity, high Ca^2+^ ions sensitivity, and response to drugs (Tricarico et al., 2004, 2005). The analysis of rat *slo1* gene at N1 and C1–C6 splice sites found the presence of 5 different variants in both fast-twitch and slow-twitch muscles, such as e17 in C1, e22, and +29 aa in C2 and rSlo27 and rSlo0 in C4 (Dinardo et al., 2012). Real time-PCR experiment showed that e22 and rSlo0 variants are markedly expressed in fast-twitch muscle, the rSlo27 is found in the slow twitch muscle giving rise to different “types” of BK channels (Dinardo et al., 2012).

In skeletal muscle, the different types of BK channel play muscle-specific roles contributing to the calcium-dependent phenotype determination/adaptation to disuse which is associated with changes of contractile properties and metabolism. After 3–14 days of muscle immobilization of the rat, in parallel with the slow-to-fast phenotype transition of the fibers, the BK channel of slow-twitch fibers acquires properties similar to those of fast-twitch fibers (Tricarico et al., 2005).

Enhanced BK channel current is observed during aging in fast-twitch fibers which are characterized by muscle disuse and fast-to slow twitch fibers transition (Tricarico et al., 1997; Pierno et al., 2014).

In addition, other than regulating fiber excitability and muscle phenotype transition during disuse, the BK channel sense extracellular K^+^ ion concentration regulating cell remodeling during hyperkalemia as observed in cell line and in a rat model of ischemia-reperfusion associated with hyperkalemia (Tricarico et al., 2002, 2013).

BK channel shows mechanosensitive properties. Stretch force can indeed induce channel activation without cytoplasmic Ca^2+^ and deletion of the Ca^2+^ bowl sequence diminishes the channel Ca^2+^ activation, but leaves the mechanosensitivity almost intact. Lack of the 59AA sequence known as STREX in the carboxyterminus domain abolished mechanosensitivity without altering Ca^2+^ activation. These evidences suggest that Ca bowl and STREX domain independently regulate BK channel activity (Zhao and Sokabe, 2008; Zhao et al., 2010). The mechanosensitivity of the BK channel may have relevance in those physiopathological conditions associated with abnormal channel function such as aging and muscle adaptation to disuse.

The presence of different types of BK channel in skeletal muscle may have implications for drug-based therapy of neuromuscular disorders, including hyper/hypokalemic periodic paralysis (PP). HypoPP is characterized by insulin-induced paralysis and hypokalemia associated with mutations of *SCN5A* and *CACNA1* genes, respectively encoding for the voltage-dependent Na^+^-channel and Ca^2+^-channel carrying abnormal H^+^/Na^+^ currents, and down-regulation of inwardly-rectifying K^+^-channels (Kir) and ATP sensitive K^+^-channel (KATP) in fast-twitch muscle (Tricarico et al., 2003a, 2008a; Jovanović et al., 2008; Tricarico and Camerino, 2011). HyperPP is associated with gain-of-function mutations of the *SCN5A* gene with persistent Na^+^ influx and depolarization, which in turn inactivates the Na^+^-channel and lead to the efflux of K^+^ ions carried by Kv/BK channels with hyperkalemia and paralysis (Cannon, 2015). KATP/BK channel openers are effective in resolving the paralytic attacks in Periodic Paralysis (Tricarico et al., 2003b, 2010; Tricarico and Camerino, 2011). Acetazolamide and dichlorphenamide act in hypoPP at micromolar concentrations of opening the BK channel in excised macropatches from fast-twitch rat fibers and are effective in repolarizing the fibers in animal models of hypoPP and in hypoPP patients (Tricarico et al., 2004; Jurkat-Rott et al., 2009; Tricarico and Camerino, 2011; Imbrici et al., 2016). In addition, acetazolamide and dichlorphenamide inhibits the membrane bound carbonic anhydrase enzymes CAIV/XIV and the CAII cytosolic form with change in the intra/extra cellular [H^+^]. This affects the activity of extra/intracellular proton exchange mechanisms. In our experiments acetazolamide inhibits the monocarboxylate transporter reducing the efflux of lactate thereby preventing myopathy (Tricarico et al., 2008b; Tricarico and Camerino, 2011). The activity of ion channels showing pH-sensitive gating may be also affected by acetazolamide and dichlorphenamide. In this respect, hypoPP patients with the histidine substitutions are responsive to the drug while those with glycine substitutions are not alleviated by lowering intracellular pH and have not benefited by acetazolamide (Tricarico and Camerino, 2011). Clinical investigation recently showed that dichlorphenamide is effective in reducing the average number of attacks per week in hypoPP patient but not in hyperPP (Sansone et al., 2016). Therefore, dichlorphenamide can be a preferential drug in hypoPP patients, including those not responsive to acetazolamide, while acetazolamide is also effective in hyperPP and myotonia. In conclusion, different factors may affect the drug responses of acetazolamide and dichlorphenamide in neuromuscular disorders. Among these, the expression of pH-sensitive mutant subunits in the muscles can play a role. Alternatively, a particular combination of BK subunits that include the slo27 may lead to the formation of BK channel unresponsive to the drugs. Drugs specifically targeting the slow-type BK channel or the fast-twitch type may be helpful in disorders affecting specific muscle phenotype.

## Author contributions

All authors listed, have made substantial, direct and intellectual contribution to the work, and approved it for publication.

## Funding

Funded by Ateneo, Univ. degli Studi di Bari, Italia 2012–14. This work was also supported by Consorzio Interuniversitario di Ricerca in Chimica dei Metalli nei Sistemi Biologici. Sede Piazza Umberto I, 1-70121-Bari, Italy

### Conflict of interest statement

The authors declare that the research was conducted in the absence of any commercial or financial relationships that could be construed as a potential conflict of interest.

## References

[B1] Cannon S. C. (2015). Channelopathies of skeletal muscle excitability. Compr. Physiol. 5, 761–790. 10.1002/cphy.c140062 25880512 PMC4754081

[B2] Contreras G. F. Castillo K. Enrique N. Carrasquel-Ursulaez W. Castillo J. P. Milesi V.. (2013). A BK (Slo1) channel journey from molecule to physiology. Channels (Austin.) 7, 442–458. 10.4161/chan.26242 24025517 PMC4042479

[B3] Dinardo M. M. Camerino G. Mele A. Latorre R. Conte Camerino D. Tricarico D. (2012). Splicing of the rSlo gene affects the molecular composition and drug response of Ca^2+^-activated K^+^ channels in skeletal muscle. PLoS ONE 7:e40235. 10.1371/journal.pone.0040235 22808126 PMC3393747

[B4] Imbrici P. Liantonio A. Camerino G. M. De Bellis M. Camerino C. Mele A.. (2016). Therapeutic approaches to genetic ion channelopathies and perspectives in drug discovery. Front. Pharmacol. 7:121. 10.3389/fphar.2016.00121 27242528 PMC4861771

[B5] Jovanović S. Du Q. Mukhopadhyay S. Swingler R. Buckley R. McEachen J.. (2008). A patient suffering from hypokalemic periodic paralysis is deficient in skeletal muscle ATP-sensitive K^+^ channels. Clin. Transl. Sci. 1, 71–74. 10.1111/j.1752-8062.2008.00007.x 20396605 PMC2854805

[B6] Jurkat-Rott K. Marc-André W. Faulera M. Guoa X. Holzherra B. D. Paczulla A.. (2009). K^+^-dependent paradoxical membrane depolarization and Na^+^ overload, major and reversible contributors to weakness by ion channel leaks. Proc. Natl. Acad. Sci. U.S.A. 106, 4036–4041. 10.1073/pnas.0811277106 19225109 PMC2644652

[B7] Pierno S. Tricarico D. Liantonio A. Mele A. Digennaro C. Rolland J. F.. (2014). An olive oil-derived antioxidant mixture ameliorates the age-related decline of skeletal muscle function. Age (Dordr.). 36, 73–88. 10.1007/s11357-013-9544-9 23716142 PMC3889891

[B8] Sansone V. A. Burge J. McDermott M. P. Smith P. C. Herr B. Tawil R.. (2016). Randomized, placebo-controlled trials of dichlorphenamide in periodic paralysis. Neurology 86, 1408–1416. 10.1212/WNL.0000000000002416 26865514 PMC4831040

[B9] Shipston M. J. (2001). Alternative splicing of potassium channels: a dynamic switch of cellular excitability. Trends Cell Biol. 11, 353–358. 10.1016/S0962-8924(01)02068-2 11514177

[B10] Toro L. Li M. Zhang Z. Singh H. Wu Y. Stefani E. (2014). MaxiK channel and cell signalling. Pflugers Arch. Eur. J. Physiol. 466, 875–886. 10.1007/s00424-013-1359-0 24077696 PMC3969412

[B11] Torres Y. P. Granados S. T. Latorre R. (2014). Pharmacological consequences of the coexpression of BK channel α and auxiliary β subunits. Front. Physiol. 5:383. 10.3389/fphys.2014.00383 25346693 PMC4193333

[B12] Tricarico D. Barbieri M. Antonio L. Tortorella P. Loiodice F. Camerino D. C. (2003b). Dualistic actions of cromakalim and new potent 2H-1,4-benzoxazine derivatives on the native skeletal muscle K ATP channel. Br. J. Pharmacol. 139, 255–262. 10.1038/sj.bjp.0705233 12770930 PMC1573836

[B13] Tricarico D. Barbieri M. Mele A. Carbonara G. Conte Camerino D. (2004). Carbonic anhydrase inhibitors are specific openers of skeletal muscle BK channel of K^+^-deficient rats. The FASEB J. 18, 760–761. 10.1096/fj.03-0722fje 14766795

[B14] Tricarico D. Camerino D. C. (2011). Recent advances in the pathogenesis and drug action in periodic paralyses and related channelopathies. Front. Pharmacol. 2:8. 10.3389/fphar.2011.00008 21687503 PMC3108473

[B15] Tricarico D. Capriulo R. Camerino D. C. (2002). Involvement of KCa^2+^ channels in the local abnormalities and hyperkalemia following the ischemia-reperfusion injury of rat skeletal muscle. Neuromuscul. Disord. 12, 258–265. 10.1016/S0960-8966(01)00270-X 11801397

[B16] Tricarico D. Lovaglio S. Mele A. Rotondo G. Mancinelli E. Meola G.. (2008b). Acetazolamide prevents vacuolar myopathy in skeletal muscle of K^+^-depleted rats. Br. J. Pharmacol. 154, 183–190. 10.1038/bjp.2008.42 18345024 PMC2438991

[B17] Tricarico D. Mele A. Calzolaro S. Cannone G. Camerino G. M. Dinardo M. M.. (2013). Emerging role of calcium-activated potassium channel in the regulation of cell viability following potassium ions challenge in HEK293 cells and pharmacological modulation. PLoS ONE 8:16. 10.1371/journal.pone.0069551 23874973 PMC3712936

[B18] Tricarico D. Mele A. Camerino G. M. Bottinelli R. Brocca L. Frigeri A.. (2010). The KATP channel is a molecular sensor of atrophy in skeletal muscle. J. Physiol. 588(Pt 5), 773–784. 10.1113/jphysiol.2009.185835 20064856 PMC2834937

[B19] Tricarico D. Mele A. Conte Camerino D. (2005). Phenotype-dependent functional and pharmacological properties of BK channels in skeletal muscle: effects of microgravity. Neurobiol. Dis. 20, 296–302. 10.1016/j.nbd.2005.03.011 16242636

[B20] Tricarico D. Mele A. Liss B. Ashcroft F. M. Lundquist A. L. Desai R. R.. (2008a). Reduced expression of Kir6.2/SUR2A subunits explains K_ATP_ deficiency in K^+^-depleted rats. Neuromuscul. Disord. 18, 74–80. 10.1016/j.nmd.2007.07.009 17825556

[B21] Tricarico D. Montanari L. Conte Camerino D. (2003a). Involvement of 3Na^+^/2K^+^ ATP-ase and Pi-3 kinase in the response of skeletal muscle ATP-sensitive K^+^ channels to insulin. Neuromuscul. Disord. 13, 712–719. 14561494 10.1016/s0960-8966(03)00095-6

[B22] Tricarico D. Petruzzi R. Camerino D. C. (1997). Changes of the biophysical properties of calcium-activated potassium channels of rat skeletal muscle fibres during aging. Pflugers Arch. 434, 822–829. 10.1007/s004240050471 9306018

[B23] Zhao H. C. Agula H. Zhang W. Wang F. Sokabe M. Lu-ming L. (2010). Membrane stretch and cytoplasmic Ca^2+^ independently modulate stretch-activated BK channel activity. J. Biomech. 43, 3015–3019. 10.1016/j.jbiomech.2010.06.018 20673577

[B24] Zhao H. C. Sokabe M. (2008). Tuning the mechanosensitivity of a BK channel by changing the linker length. Cell Res. 18, 871–878. 10.1038/cr.2008.88 18663377

